# Rapid formation and rupture of an infectious basilar artery aneurysm from meningitis following suprasellar region meningioma removal: a case report

**DOI:** 10.1186/s12883-020-01673-9

**Published:** 2020-03-14

**Authors:** Xu Wang, Ge Chen, Mingchu Li, Jiantao Liang, Hongchuan Guo, Gang Song, Yuhai Bao

**Affiliations:** Department of Skull Base Surgery Center, Department of Neurosurgery, XuanWu Hospital, Capital Medical University, Beijing, China

**Keywords:** Infectious intracranial aneurysms, Bacterial meningitis, Endoscopic transnasal operation, Septic microemboli

## Abstract

**Background:**

Infectious basilar artery (BA) aneurysm has been occasionally reported to be generated from meningitis following transcranial operation. However, infectious BA aneurysm formed by intracranial infection after endoscopic endonasal operation has never been reported.

**Case presentation:**

A 53-year-old man who was diagnosed with suprasellar region meningioma received tumor removal via endoscopic endonasal approach. After operation he developed cerebrospinal fluid (CSF) leak and intracranial infection. The patient ultimately recovered from infection after anti-infective therapy, but a large fusiform BA aneurysm was still formed and ruptured in a short time. Interventional and surgical measures were impossible due to the complicated shape and location of aneurysm and state of his endangerment, therefore, conservative anti-infective therapy was adopted as the only feasible method. Unfortunately, the aneurysm did not disappear and the patient finally died from repeating subarachnoid hemorrhage (SAH).

**Conclusion:**

Though extremely rare, it was emphasized that infectious aneurysm can be formed at any stage after transnasal surgery, even when the meningitis is cured. Because of the treatment difficulty and poor prognosis, it was recommended that thorough examination should be timely performed for suspicious patient to make correct diagnosis and avoid fatal SAH.

## Background

As a peculiar type of aneurysm, infectious intracranial aneurysms (IIAs) account for only about 1–6% of all intracranial aneurysms [1, 2], most of which are secondary to infectious endocarditis [3–5]. According to the systematic review conducted by Ali Alawieh et al., nearly 70% of all IIAs are resulted from infectious endocarditis [6]. Occasionally, some other infectious factors such as bacterial meningitis and cavernous thrombophlebitis can also lead to IIAs [4–7]. Compared with other conventional intracranial aneurysms, IIAs usually predict worse prognosis because of higher aneurysmal rupture rate [5]. The systematic review by Amit Singla et al. in 2015 revealed that the mortality rate of IIAs is 26.7% after conservative treatment and 15.1% after surgical or interventional therapy [8].

Most IIAs are located at distal branches of anterior circulation, with posterior circulation rarely involved. According to sporadic case reports about IIAs of vertebrobasilar artery, they were most commonly secondary to infection following transcranial surgery. However, infectious BA aneurysm formed after endoscopic endonasal operation has never been reported. In this study, it was reported that a rare case of infectious BA aneurysm secondary to intracranial infection after removal of meningioma in suprasellar region via endoscopic endonasal approach. The peculiarity of current case lies in the fact that the aneurysm is originated even after recovery of infection. Afterwards, a systematic review of relative literatures was performed.

## Case presentation

**Fig. 1:**
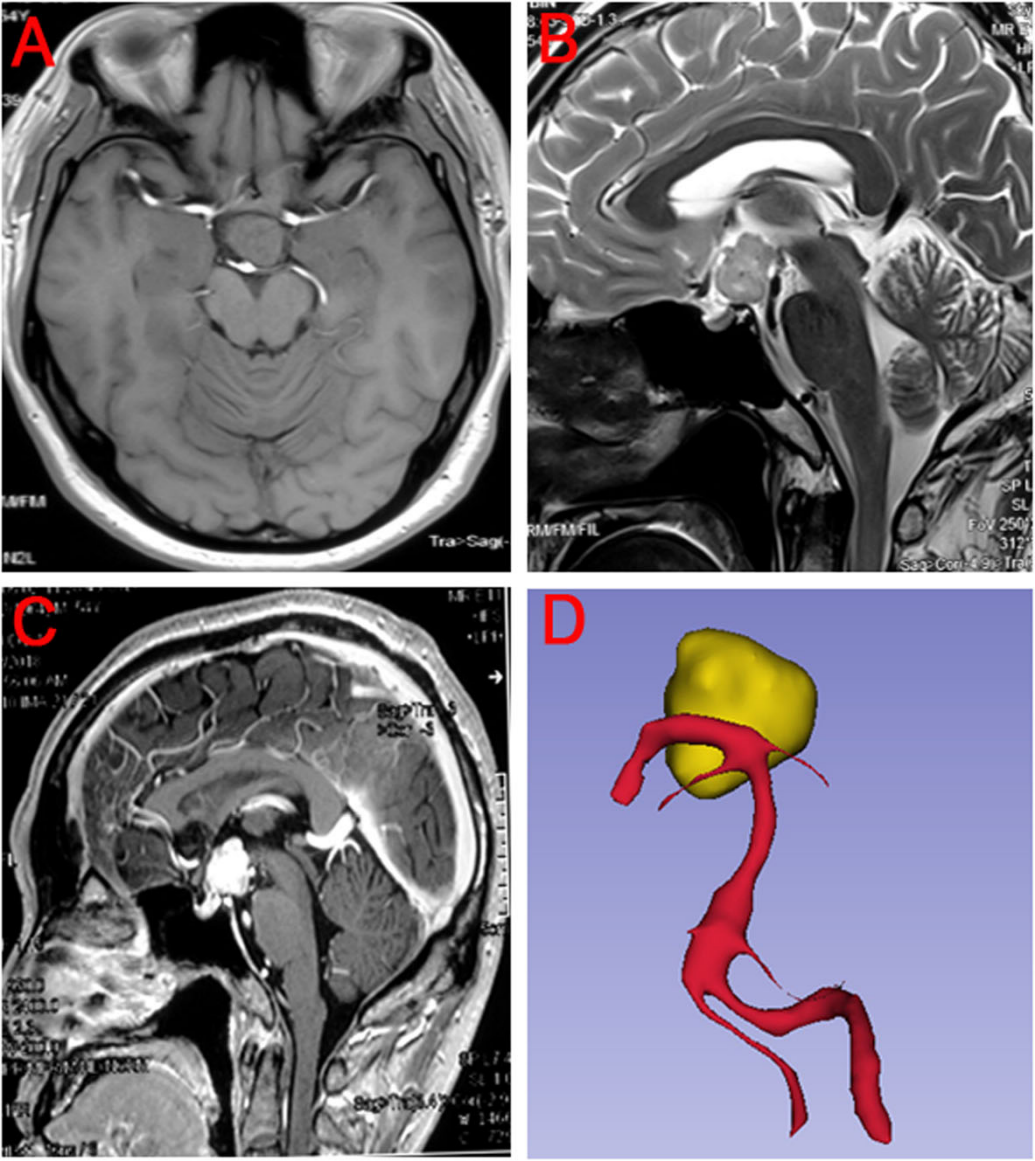
Preoperative brain MRI. **a** Axial T1WI MRI before the first operation showed an isointense lesion located at suprasellar region which was regular in shape and near the BA apex and PCA. **b** Sagittal T2WI MRI showed the lesion was isointense and closely related to the third ventricle. Moreover, there was no abnormity of BA. **c** Sagittal contrast-enhanced T1WI MRI showed the tumor was homogeneously enhanced and the BA was also shown. **d** Three dimensional-reconstructed image from preoperative MRI showed no aneurysm at BA and its branches

A 53-year-old male presenting with paroxysmal headache was admitted to our hospital. Brain magnetic resonance imaging (MRI) showed an oval tumor located at suprasellar region, neighboring to BA (Fig. 1a-c). Three dimensional-reconstructed image from preoperative MRI showed that the BA and its branches were normal (Fig. 1d). The patient was diagnosed as meningioma based on the preoperative MRI. Surgical removal of tumor was performed via endoscopic endonasal approach. He developed CSF rhinorrhea 17 days after operation and endoscopic repair was timely conducted the next day.

On the second day after repair operation, he developed fever and neck rigidity. The lumbar puncture showed an opening pressure as high as 33 cm H_2_O, glucose of CSF was 0.36 mg/dL, protein of CSF was 213 mg/dl, and white blood cell count was 19223*10^6 cells/L with 81% polymorphonuclear granulocyte. Intracranial infection was diagnosed based on the laboratory report and clinical symptoms. Whereas, the CSF bacterial culture result was negative. Meropenem and vacocin vancomycin were both intravenously and intrathecally used for anti-inflammatory treatment. The intracranial infection got significant improvement after 2 weeks of anti-inflammatory therapy.

**Fig. 2:**
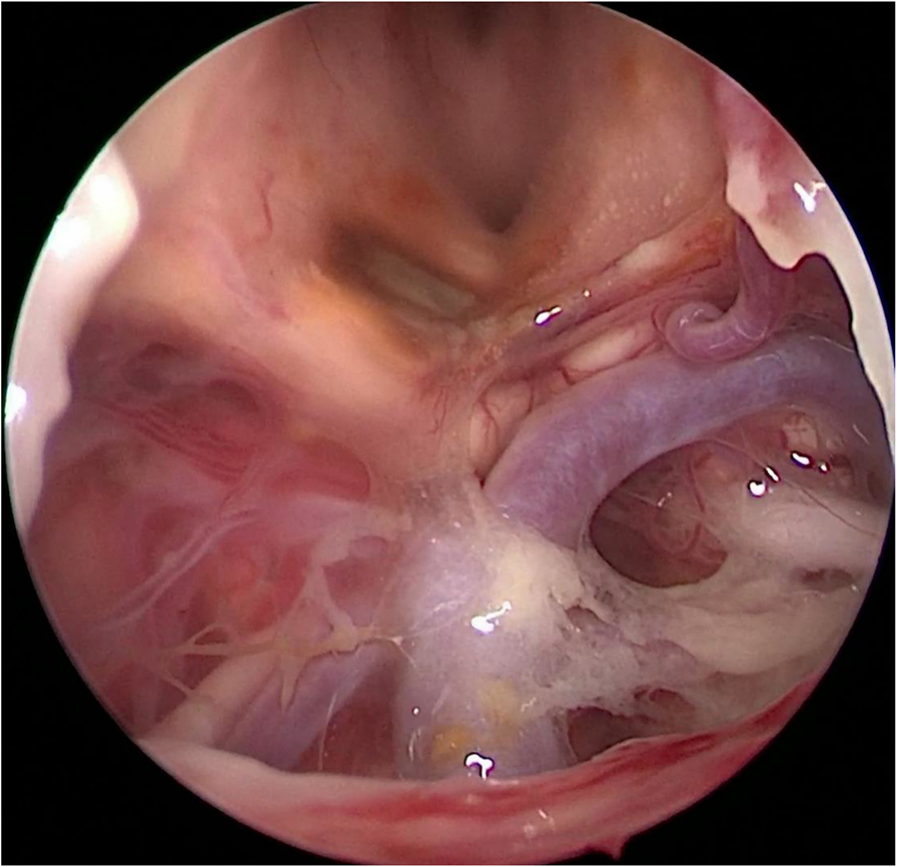
Intraoperative photograph from the second repair operation clearly showed bilateral SCA, PCA, oculomotor nerves, and BA. No fusiform aneurysm was observed at BA, but surface of BA and left SCA was covered with many infectious floccules

**Fig. 3:**
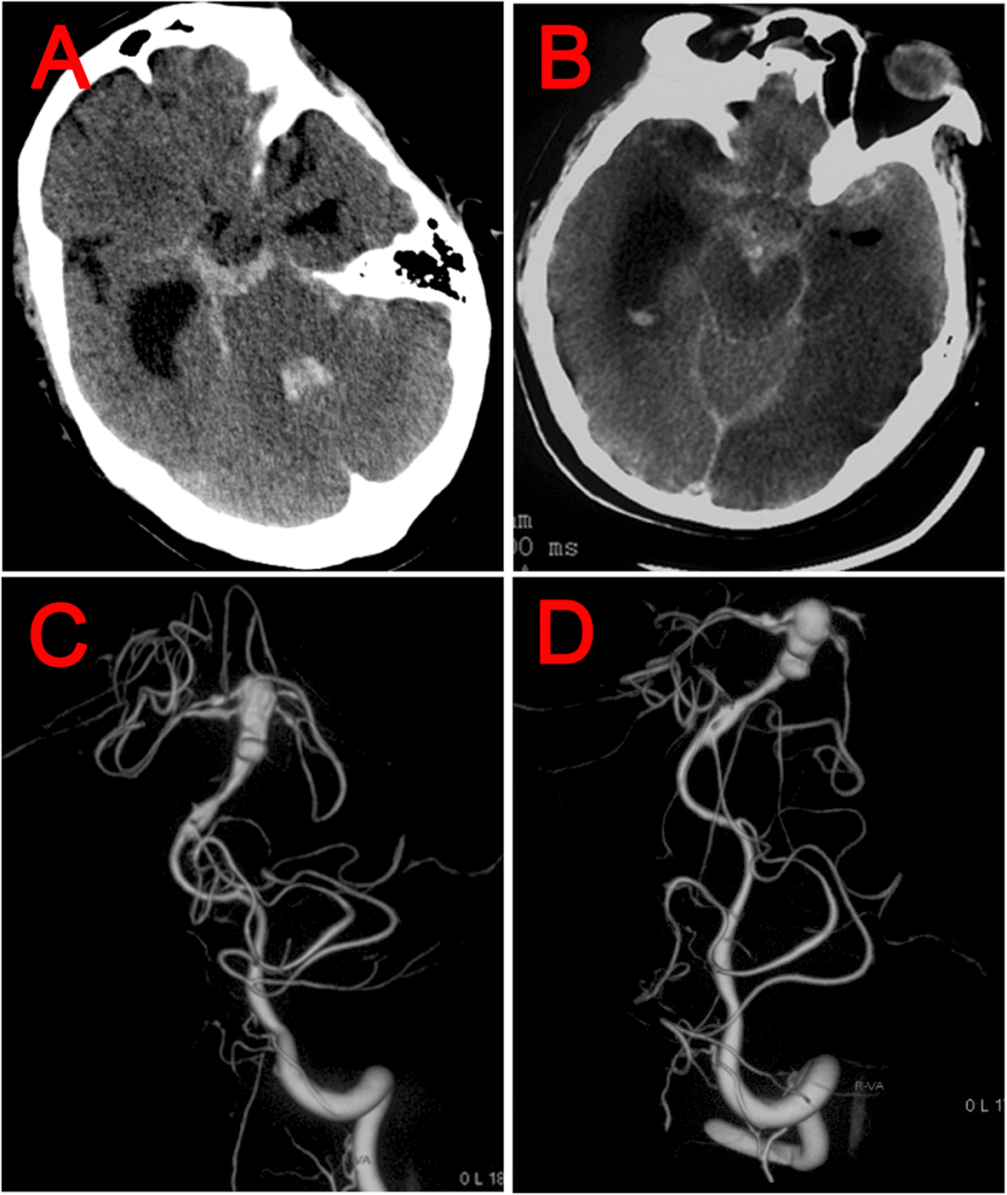
**a** Brain CT scan performed immediately after attack of sudden severe headache and loss of consciousness showed SAH at prepontine cistern and fourth ventricle. **b** Brain CT rechecked in the next day after SAH showed SAH was aggravated. **c-d** DSA showed an enormous fusiform aneurysm at the trunk of BA, which gave rise to some aneurismal secondary cysts. Moreover, original sites of bilateral SCA and PCA were also involved

At the 15th day after the first repair operation, CSF rhinorrhea was confirmed to continue and once again, endoscopic repair for CSF leak was performed. BA and its branches were exposed during the second repair operation and no abnormity was shown, nevertheless a lot of infectious floccules surrounding the trunk of BA were clearly revealed (Fig. 2). During the whole surgery, BA was not touched by any surgical instruments, so traumatic injury was avoided. Whereafter, intracranial infection was completely healed but the patient still developed headache and gradually aggravated consciousness disturbance. The patient developed sudden severe headache and loss of consciousness 12 days after the second repair operation. Brain computerized tomography (CT) scan revealed hydrocephalus and SAH predominantly at prepontine cistern (Fig. 3a). Physical examination showed the dilation of left pupil and disappearance of light reaction. Urgent tracheal intubation and ventricle puncture and drainage were performed. Twenty-four hours later, the right pupil also dilated and lost the light reaction. Brain CT rechecked showed SAH was aggravated (Fig. 3b).

Digital substraction angiography (DSA) showed a large fusiform aneurysm at BA (Fig. 3c-d). It was believed the aneurysm was formed by spread of contiguous infection into arterial wall. DSA revealed remarkable dilatation and tortuosity of BA, which gave rise to some aneurismal secondary cysts. Moreover, original sites of bilateral superior cerebellar artery (SCA) and posterior cerebral artery (PCA) were also involved. Coupled with the state of his endangerment (Hunt-Hess grade 5) and infectious environment, both open surgery and interventional therapy were impossible and only the conservative anti-inflammatory treatment was continued. Ultimately, the aneurysm did not disappear and he died from repeating SAH 17 days later.

We adhered to CARE guidelines/methodology.

## Discussion and conclusions

IIAs are very rare, 70–80% of which are secondary to left-sided bacterial endocarditis [4, 9, 10]. Ferdinand K Hui et al. retrospectively analyzed 168 patients with infectious endocarditis who received DSA, revealing that 8.9% of all patients are concomitant with IIAs [11]. On the other hand, intracranial infection following craniocerebral operations is another important source of IIAs [1, 7, 12–14]. Other more rare reasons of IIAs include orbital cellulitis, bacterial pneumonia, osteomyelitis of the skull and sinus infections [6, 15].

According to literature reports, viridans group streptococci and *Staphylococcus aureus* is the most common pathogenic bacteria, which is responsible for 57 to 91% of all IIAs [15–17]. As no positive results were obtained from the blood and CSF culture in the patient, meropenem and vacocin vancomycin were empirically adopted both intravenously and intrathecally for the anti-inflammatory treatment.

Generally speaking, IIAs are formed by inflammation and destruction of intracranial arterial walls. Bacterial embolus from primary infection can arrive at the vasa vasorum of normal intracranial arteries, leading to the occlusion of the vasa vasorum and intense inflammation in the adventitia and muscle layer, which then resulted in weakness of intracranial arterial walls and formation of aneurysm dilation. IIAs are always pseudoaneurysms because the muscular layer is usually involved [15, 18].

Among patients with bacterial meningitis, IIAs are believed to be caused by dispersion of septic microemboli into nearby intracranial arteries. As for the current patient, no abnormity of BA and bilateral PCA other than some infectious floccules around the trunk of BA was shown during the second repair operation (Fig. 2), intraoperative traumatic injury of BA was also excluded. Whereas, the patient developed symptoms of SAH a few days later. Therefore, it was believed that the aneurysm was infectious in origin and formed and ruptured in a short period after the second repair operation.

The natural history of IIAs is still under debate. Kang-Ho Choi reported a patient with IIA from bacterial meningitis, which was formed within 24–48 h from the spread of infection [19]. Robert M. Koffie once reported a special case of IIA, who died of enlargement and rupture of middle cerebral artery (MCA) aneurysm within only 24 h [20]. Previous animal model showed that IIAs can be formed within 24 h since the drop of inflammatory embolus [18]. Our patient developed intracranial infection after CSF leak from suprasellar cistern, then the nearby BA was invaded by inflammatory embolus from surgical field and fusiform aneurysm was formed within a short time. It was believed that the BA aneurysm was formed within a few days from second repair operation to the attack of SAH. It is very strange that in current patient the aneurysm was formed after recovery of intracranial infection, which has been rarely reported.

IIAs secondary to infectious endocarditis are often peripheral to the first bifurcation of a intracranial artery in the anterior circulation [21, 22], with distal branches of MCA most commonly involved [3, 10, 23, 24]. On the other hand, IIAs induced by bacterial meningitis are frequently localized to more proximal regions of intracranial arteries [1, 9, 25, 26]. All in all, most IIAs are located at the anterior circulation regardless of the infection source. A systematic review including 86 patients from 11 studies indicated that 93.2% of those IIAs are located at anterior circulation, while only 6.8% are located at posterior circulation [27]. CSF leak and intracranial infection are common after sellar region tumor removal via the endoscopic endonasal approach. However, it is extremely rare that IIAs can be formed following endoscopic operation. To our knowledge, this is the first report of a large infectious BA aneurysm secondary to CSF leak and intracranial infection after endoscopic transnasal surgery.

Other than anterior circulation IIAs that are often induced by bacterial endocarditis, IIAs of vertebro-basilar artery are more commonly secondary to bacterial meningitis, with higher mortality rate [1]. A systematic review revealed that IIA at vertebro-basilar artery (vertebrobasilar junction) was observed in only one case among all 62 patients. This case received endovascular balloon occlusion treatment and retained with disabling stroke [25]. Daniel *L. barrow* reported two cases of BA IIAs in 1990. One case was induced by fungal infection after craniotomy and died of aneurysm rupture. The other 12-year-old patient developed proximal BA IIA secondary to bacterial meningitis, and recovered well after surgical clipping of aneurysm [13]. Currently, the largest single center sample of BA IIAs has been reported by Sudheeran Kannoth et al. Among 25 patients with 29 IIAs in the study, all three patients with BA IIAs died of aneurysm rupture at last [1].

Due to the unique formation mechanism and morphological characteristics, the therapeutic strategy of IIAs is different from that of other regular aneurysms. It has been widely believed that IIAs should be individually treated based on whether the aneurysm ruptures or not, the location of aneurysm, whether the parent artery supplies the eloquent brain tissue or not. Some unruptured aneurysms can be resolved with the antibiotic therapy, but fatal rupture and bleeding can also occur during the conservative treatment [22]. It is still controversial for the treatment of unruptured IIAs [1, 4, 28, 29]. Some doctors recommend that besides the antibiotic therapy, active surgical or interventional treatment is needed for all unruptured IIAs in consideration of the high rupture and mortality rates [2, 17].

Interventional measures including direct aneurysm occlusion in more proximal locations and parent vessel occlusion in distal lesions are suitable for those IIAs not accompanied with evident intracranial hematoma [25]. More and more IIAs can be well controlled by the interventional therapy in recent years. It has been reported that embolization by Onyx is a safe and effectual treatment option for some IIAs [30, 31]. Geoffrey Appelboom reported that a patient with IIA at cavernous segment ICA secondary to meningitis is successfully treated by flow-diverting stent graft [7].

In some cases, interventional therapy can result in ischemic complications. If rupture of IIAs results in severe intracranial hematoma and space-occupying effect, interventional therapy will not be suitable. By the opening surgery parent vessel sacrifice and aneurysm clipping could be performed in addition to evacuation of hematoma. Ruptured IIAs of the distal MCA could be also treated by trapping and bypass surgery [32]. Nussbaum et al. reported that, good outcomes can be achieved for some complex PCA or basilar aneurysm by low-flow or high-flow bypass treatment [33].

In our patient, the aneurysm was large and involved in the original sites of bilateral SCA and PCA. Basilar sacrifice with bypass might have saved his life, but it was not conducted for some reasons at last. Firstly, severe SAH developed in a short time at this patient. When basilar aneurysm was confirmed by DSA, the patient was in a very danger condition (Hunt-Hess grade 5). In consideration of his state of endangerment, it was believed that he could hardly tolerate aggressive bypass operation. Secondly, because of previous severe intracranial infection, basilar artery and bilateral PCA were surrounded by a lot of infectious floccules. It was assumed that it was not feasible to perform bypass operation under this environment. Therefore, conservative anti-inflammatory treatment was adopted as the only feasible method. Unfortunately, the aneurysm did not disappear and resulted in fatal SAH.

IIA of BA is a very rare and dangerous complication from meningitis after endoscopic endonasal surgery, which can be formed and enlarged in a short time even after recovery from intracranial infection. Because of the treatment-difficulty and poor-prognosis, it was recommended that thorough examination should be timely performed for suspicious patient to make correct diagnosis and avoid fatal SAH.

## Data Availability

All data analyzed during this study are included in this published article.

## Abbreviations

BA: Basilar artery
CSF: Cerebrospinal fluid
CTA: Computed tomography angiography
DSA: Digital subtraction angiography
ICA: Internal carotid artery
IIA: Infectious intracranial aneurysm
IIAs: Infectious intracranial aneurysms
MCA: Middle cerebral artery
MRI: Magnetic resonance imaging
PCA: Posterior cerebral artery
SAH: Subarachnoid hemorrhage
SCA: Superior cerebellar artery
T1WI: T1-weighted imaging
T2WI: T2-weighted imaging
